# Perceived Physical Fatigability Improves After a Weight Management Intervention

**DOI:** 10.1093/geroni/igaa057.3080

**Published:** 2020-12-16

**Authors:** Jessica Graves, Theresa Gmelin, Robert Boudreau, Steven Albert, Anne Newman, Elizabeth Venditti, Nancy Glynn

**Affiliations:** University of Pittsburgh, Pittsburgh, Pennsylvania, United States

## Abstract

The effects of a weight loss and physical activity (PA) intervention on improving perceived physical fatigability are unknown. We examined this question in a subset (n=79) of older adults who are obese enrolled in the 13-month Mobility and Vitality Lifestyle Program (mean□SD age 68.8±4.2 years, 83.5% female, 26.6% African American, body mass index 34.6±4.3 kg/m2). Accelerometer-assessed PA (mean/day vector magnitude) was measured with a wrist-worn triaxial GT3X+ ActiGraph for 7 full days. Perceived physical fatigability was measured using the 10-item self-administered Pittsburgh Fatigability Scale (PFS, 0-50; lower score= less fatigability). Baseline PFS was 18.7±8.5 with 69.6% having higher fatigability (PFS ≥15). At 13-months, PFS decreased by 15% (2.8 points) to 15.9±8.4 (p<0.01) and prevalence of higher fatigability declined to 60.8%. Concurrently, participants lost 6.2% of their body weight and PA increased by 2.4%. A lifestyle intervention may be effective at reducing fatigability, an important component in the age-related disablement pathway.

